# Quantitative Determination of Steroidal Saponins of *Semen Allii Fistulosi* Using HPLC-ELSD and HPLC-MS

**DOI:** 10.1155/2024/1872918

**Published:** 2024-11-01

**Authors:** Zhancai Zheng, Deduo Xu, Wenquan Lu, Wansheng Chen, Yingbo Yang, Zhijun Wu

**Affiliations:** ^1^Department of Pharmacy, Second Affiliated Hospital of Naval Medical University, Shanghai 200003, China; ^2^Research and Development Center of Chinese Medicine Resources and Biotechnology, The Ministry of Education (MOE) Key Laboratory for Standardization of Chinese Medicines, Institute of Chinese Materia Medica, Shanghai University of Traditional Chinese Medicine, Shanghai 201203, China; ^3^R&D Center, Jiangsu Kanion Pharmaceutical Co., Ltd., Lianyungang 222001, China

## Abstract

In this study, high-performance liquid chromatography-evaporative light scattering detection (HPLC-ELSD) and HPLC-mass spectrometry (HPLC-MS) were employed for the quantitative analysis of steroidal saponins of *Semen Allii Fistulosi*. The HPLC-ELSD method was simple, accurate, and repeatable, and the recoveries were 97.91%∼99.77%. Similarly, the HPLC-MS method was rapid, sensitive, accurate, and reproducible, and the recoveries ranging from 95.61% to 99.70% in the control samples. The sensitivity of the HPLC-MS method was several times higher than that of the HPLC-ELSD method. These results collectively demonstrated that these methods are suitable for the content determination of steroidal saponins of *Semen Allii Fistulosi* and the identification of its plant source.

## 1. Introduction

Myocardial ischemia (MI) is a prevalent cardiovascular disease characterized by a high incidence. At present, several classes of common antimyocardial ischemia drugs have been licensed, encompassing nitrates, *β*-blockers, calcium antagonists, as well as adjunctive therapy drugs such as thrombolytic drugs, platelet aggregation inhibitors, and lipid-lowering drugs. However, the aforementioned drugs have limitations and side effects. For instance, thrombolytic drugs, and antiplatelet can increase the risk of bleeding. Nitrates and calcium antagonists are not recommended in hypotensive patients. Moreover, prolonged use of *β*-blockers can impair glucose tolerance, whilst lipid-lowering drugs must be administered over an extended period to be effective. Therefore, research on antimyocardial ischemia drugs is predominantly focused on protecting myocardial cells by enhancing the metabolism of ischemic myocardial cells and their ability to resist hypoxia injury [1].

*Allium fistulosum* (Welsh onion) is extensively cultivated in China, Japan, and Korea. *A. fistulosum* is a traditional Chinese vegetable and is one of the most commonly used vegetables in the daily life of Chinese families. *A*. *fistulosum* is an popular household ingredient in Eastern countries and is considered a good source of nutrients. It possesses various biological activities, such as antibacterial, antiplatelet, antihypercholesterolemic, anticancer, antiobesity, *α*-glucosidase inhibitory, antioxidant, anti-inflammatory, and antihypertensive properties [2–11]. Its chemical constituents reported include steroidal saponins, steroidal sapogenins, proteins, sulfur-containing compounds, fatty acids, and phenolic acids [12–19]. Its biological activities are attributable to its phytochemicals. For example, the antioxidant capacity of *A. fistulosum* is highly correlated with its total phenolic content, whereas its antibacterial activity is contributable to allicin [20]. Flavonoids in *A*. *fistulosum*, including quercetins and quercetin glycosides, have been reported to be associated with its anticancer and antioxidant properties [5, 21]. Traditional Chinese medicine has recognized the therapeutic effects of the bulbs and roots of *A. fistulosum* for febrile conditions, headaches, abdominal pain, diarrhea, snakebites, ocular disorders, and habitual abortion [22].

In recent years, the impact of Allium focused on cardiovascular effects has garnered extensive attention [23–28]. Our previous study on the seeds of *A. fistulosum* extract (SAFE) demonstrated that SAFE can significantly alleviate MI severity, the myocardial infarction, and injury to ischemic myocardial cells. Furthermore, it can inhibit LDH and CK overflow and concomitantly limit their activity of LDH and CK in serum of rats and dogs. Steroidal saponins were reported to be responsible for the cardiovascular activity [29].

*A. fistulosum* is rich in resources, and widely distributed in most areas of China. Steroidal saponin content in *A. fistulosum* is affected by the environment, soil, climate and other factors. *Semen Allii Fistulosi* is the seeds of *A. fistulosum.* In traditional Chinese medicine, *Semen Allii Fistulosi* is used for the treatment of renal deficiency, dizziness, cold, and as a tonic and aphrodisiac [17, 30, 31]. Currently, there are only a few literature reported about qualitative analytical methods to determine the steroidal saponins of Allium [32–34], and limited studies for the quantitative analysis of steroidal saponins in Allium have been reported [35]. The quantitative analysis of steroidal saponins remains a challenge for the analytical community. Therefore, an accurate and reliable method is urgently needed for the quantitative determination of the steroidal saponins of Allium. In the current study, a simple, accurate, and reliable analytical method using high-performance liquid chromatography-evaporative light scattering detection (HPLC-ELSD) and a HPLC-mass spectrometry (HPLC-MS) were established for the quantitative determination of steroidal saponin of *Semen Allii Fistulosi*. The developed methods are simple and ideal for the routine analysis of steroidal saponins in Allium and quality control.

## 2. Materials and Methods

### 2.1. Equipment and Materials

**Figure 1:**
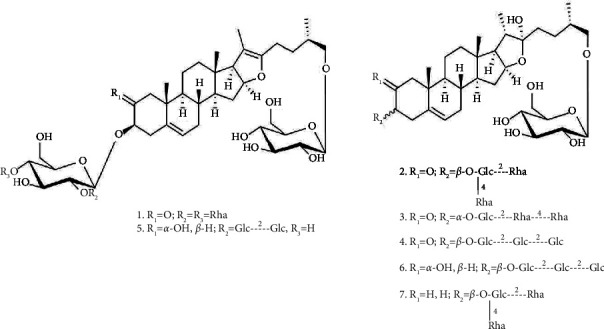
The chemical structures of 7 reference substances. (1) Fistulosaponin A; (2) Fistulosaponin B; (3) Fistulosaponin C; (4) Fistulosaponin D; (5) Fistulosaponin E; (6) Fistulosaponin F; (7) LW-24.

The experimental research and field studies, including the collection of plant materials, were conducted in accordance with institutional, national, and international guidelines and legislation. The seeds of *A. fistulosum* were collected in different parts of China and were authenticated by Prof. Han-Ming Zhang of the Department of Pharmacognosy, School of Pharmacy, Naval Medical University, Shanghai, China. Fistulosaponin A, B, C, D, E, F, and 26-O-*β*-D-glucopyranosyl-(25*R*)-furost-5-ene-3*β*,22,26-triol-3-O-2,4-di-O-(*α*-L-rhamnopyranosyl)-*β*-D-glucopyranoside (LW-24) (Figure 1), were isolated from *Semen Allii Fistulosi*, and the structures of the compounds were subsequently characterized via spectroscopic analyses (UV, IR, MS, ^1^H NMR, ^13^C NMR, 2D NMR) and chemical methods. Their purity exceeded 98%, as determined by the HPLC peak area normalization method [12].

The analyses were carried out employing the Agilent 1100 HPLC equipped with a SEDEX 85LT-ELSD detector and Agilent G1946D quadrupole mass spectrometer featuring an electrospray ion source.

### 2.2. Quantitative Analysis of Steroidal Saponins With HPLC-ELSD

#### 2.2.1. Liquid Chromatographic Conditions

HPLC column (Diamonsil C18, 5 *μ*m, 4.6 × 250 mm, Dikma); the mobile phases were composed of solvents A (water and formic acid, adjusted pH: 3.3) and B phase (acetonitrile). The gradient elution program was as follows: A, 100%–78% at 0–3 min, 78%–77% at 3–16 min, 77%–73% at 16–40 min, and 73%–10% at 40–50 min. The column temperature was maintained at 30°C, with a flow rate of 1.0 mL·min^−1^. The injection volume was 20 *μ*L. Lastly, ELSD detector parameters were set as follows: drift tube temperature was maintained at 40°C, and carrier gas pressure was set to 3.5 Bar.

#### 2.2.2. Preparation of Reference Solution*s*

Fistulosaponin B, D, F, and LW-24 were weighed at 35.20 mg, 30.80 mg, 11.75 mg, and 16.80 mg, dissolved in 30% acetonitrile-water solution, and fixed in a 10 mL volumetric flask to prepare the reference solutions, respectively.

#### 2.2.3. Preparation of Sample Solutions

5 g powder seeds of *A. fistulosum* were accurately weighed and transferred into a 100 mL Erlenmeyer flask, which was then sealed with a plug. Next, 10 mL of pure water was added, and the mixture was allowed to soak overnight. Following this, the mixture was subjected to ultrasonic treatment for 30 min, after which the residue was filtered using 10 mL pure water. The above-mentioned step was repeated. Following this, the container and residue were washed with pure water, and the resulting extract was inserted into a 100 mL flask. Afterward, the extract was concentrated under reduced pressure and placed in a 1 mL volumetric flask, and the volume was adjusted to 1 mL using 30% acetonitrile-water solution. After mixing and filtration through a 0.45 *μ*m microporous membrane, the filtrate was obtained as the sample solution.

### 2.3. Quantitative Analysis of Steroidal Saponins With HPLC-MS

#### 2.3.1. Liquid Chromatographic Conditions

HPLC column (Agilent Eclipse XDB C18, 3.5 *μ*m, 2.1 × 100 mm, Dikma); the mobile phases consisted of solvent A (water) and B (acetonitrile). The gradient elution program was as follows: A, 90%–82% at 0–5 min, 82%–79% at 5–15 min, 79%–76% at 15–18 min, 76%–75% at 18–25 min, 75%–40% at 25–30 min, 40%–10% at 30–35 min. The column temperature was maintained at 30°C, with a flow rate and injection volume of 0.3 mL·min^−1^ and 5 *μ*L, respectively.

#### 2.3.2. MS Conditions

An ESI ion source in the negative ionization mode was used for mass spectrometry analysis. Flow injection analysis (FIA) was utilized to optimize the working parameters of mass spectrometry. The conditions were as follows: ion source temperature: 350°C, dry gas flow rate: 12 L·min^−1^, atomizing gas pressure: 40 psi, debris voltage: 70 v, ion source voltage: 4000 V.

#### 2.3.3. Preparation of Reference Solutions

Fistulosaponin B, D, F, and LW-24 were accurately weighed at 21.72 mg, 15.32 mg, 9.19 mg, and 3.09 mg, respectively, and then dissolved in 30% acetonitrile-water solution and fixed in 50 mL volumetric flask. At the same time, Fistulosaponin A, C, and E were accurately weighed at 1.96 mg, 2.46 mg, and 2.37 mg, respectively, dissolved in 30% acetonitrile-water solution, and fixed in a 10 mL volumetric flask.

#### 2.3.4. Preparation of Sample Solutions

1 g powder seeds of *A. fistulosum* was accurately weighed and transferred into a 100 mL Erlenmeyer flask and sealed with a plug. 10 mL of pure water was thereafter added, and the mixture was allowed to soak overnight. It was then subjected to ultrasonic treatment for 30 min two times, followed by filtration. The resulting extract was transferred to a 10 mL volumetric flask, and its volume was adjusted to 10 mL using 30% acetonitrile-water solution. After mixing, the solution was filtered through a 0.25 *μ*m microporous membrane to obtain the filtrate as the sample solution.

### 2.4. Method Performance [36]

#### 2.4.1. Linearity, Limit of Detection (LOD), and Limit of Quantification (LOQ)

To establish calibration curves, at least five predefined concentrations of standard stock solutions were prepared by serially diluting the standard solution with appropriate volumes of solvent, and all operations were performed in triplicates. All calibration curves were plotted based on linear regression analysis of the integrated peak area (*y*) versus concentrations (*x*, *μ*g/mL) in the standard solution at five different concentrations. The LOD was calculated at a signal-to-noise ratio of 3, whilst the LOQ was determined as the lowest concentration in the linear range of each analyte.

#### 2.4.2. Precision, Repeatability, Stability, and Recovery

The two methods, namely HPLC-ELSD and HPLC-MS, were validated through precision, repeatability, stability, and recovery assessments. Specifically, precision was evaluated using six interday and intraday relative standard deviations (RSD). The repeatability of the method was determined through the extraction and analysis of six replicates of *Semen Allii Fistulosi* samples. The stability of the samples was examined at predetermined intervals of 0, 1, 2, 4, 8, 12, and 24 h, with RSD determined for the detected concentrations of each analyte. Method accuracy was tested through spiking experiments. The recovery test was performed by introducing standard solutions at low, medium, and high levels. Recovery was calculated using the following equation: recovery = (total detected amount − original amount)/added amount × 100%.

## 3. Results and Discussion

### 3.1. HPLC-ELSD Results

#### 3.1.1. Methodological Investigation

Table 1 summarizes the regression equations, *R*^2^ values, and linear ranges for the 4 steroidal saponins. Linear correlations were observed between the peak areas and concentrations for each analyte. Moreover, the calibration curves demonstrated satisfactory linearity (*R*^2^ ≥ 0.999) in the tested concentration ranges. The LOD and LOQ for the 4 steroidal saponins were 5.25–6.08 *μ*g/mL and 17.50–20.27 *μ*g/mL, respectively, implying that the calibration curves were within adequate ranges. Taken together, the developed method exhibited robust sensitivity for the separation and analysis of the 4 steroidal saponins.

#### 3.1.2. Precision, Repeatability, Stability, and Recoveries

The results revealed (Table 2) that variations (RSD) for the interday and intraday analyses were 1.54%–2.48% and 1.53%–2.02%, with a repeatability of 0.72%–2.44% and a stability of 1.28%–2.16%. The recovery rates ranged from 98.24% to 99.08%, with RSD values of 1.52%–1.78%. These results collectively highlighted the precision, repeatability, and stability of the method for the determination of the 4 steroidal saponins present in *Semen Allii Fistulosi* samples.

#### 3.1.3. Sample Analysis

**Figure 2:**
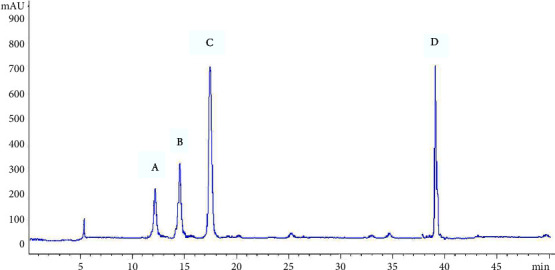
HPLC-ELSD chromatogram for the 4 steroidal saponin mixed standards ((A) Fistulosaponin F; (B) Fistulosaponin D; (C) Fistulosaponin B; (D) Lw-24).

**Figure 3:**
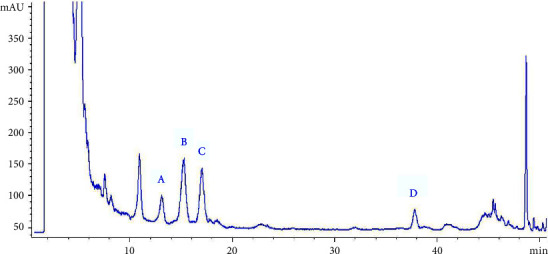
HPLC-ELSD chromatogram for the sample from Zigong, Sichuan ((A) Fistulosaponin F; (B) Fistulosaponin D; (C) Fistulosaponin B; (D) Lw-24).

The contents (*n* = 3) of the 4 steroidal saponins in *Semen Allii Fistulosi* samples were calculated using an external standard method derived from their respective calibration curves, as listed in Table 3. The HPLC chromatograms are illustrated in Figures 2 and 3.

### 3.2. HPLC-MS Results

#### 3.2.1. Methodological Investigation

All calibration curves were constructed via linear regression analysis of the integrated peak areas (*y*) versus concentrations (*x*, *μ*g/mL) of the 7 marker constituents in the standard solution at six different concentrations. The regression equations, correlation coefficients, and linear ranges for the analysis of the 7 marker constituents are detailed in Table 4. As anticipated, the calibration curves displayed good linearity (*R*^2^ ≥ 0.999) in the tested concentration ranges. Meanwhile, the LOD and LOQ for the 7 compounds were 0.12–0.90 *μ*g/mL and 0.38–1.77 *μ*g/mL, respectively. Overall, the developed method exhibited good sensitivity for the analysis of steroidal saponins of Fistulosaponin A, B, C, D, E, F, and Lw-24.

#### 3.2.2. Precision, Repeatability, Stability, and Recovery

Under the above-mentioned MS conditions, the variations (RSD) for the interday and intraday analyses were 1.21%–2.09% and 0.77%–1.75%, with a repeatability of 2.96%–4.65% and a stability of 0.92%–3.70%, as presented in Table 5. The recovery ranged from 96.87% to 99.23%, whilst the RSD values ranged between 0.87% and 2.41%. These results conjointly highlighted the high precision, repeatability, and stability of the method for the determination of the 7 steroidal saponins in *Semen Allii Fistulosi* samples.

#### 3.2.3. Sample Analysis

**Figure 4:**
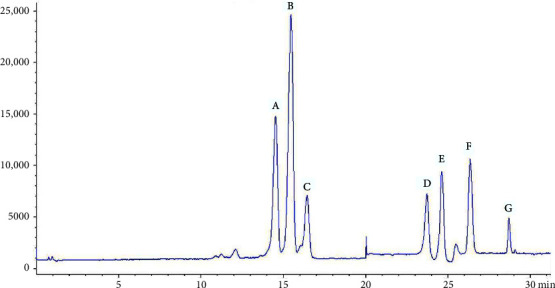
HPLC chromatograms of the 7 steroidal saponin mixed standards ((A) Fistulosaponin F; (B) Fistulosaponin D; (C) Fistulosaponin B; (D) Lw-24; (E): Fistulosaponin C; (F) Fistulosaponin E; (G) Fistulosaponin A). HPLC, high-performance liquid chromatography.

**Figure 5:**
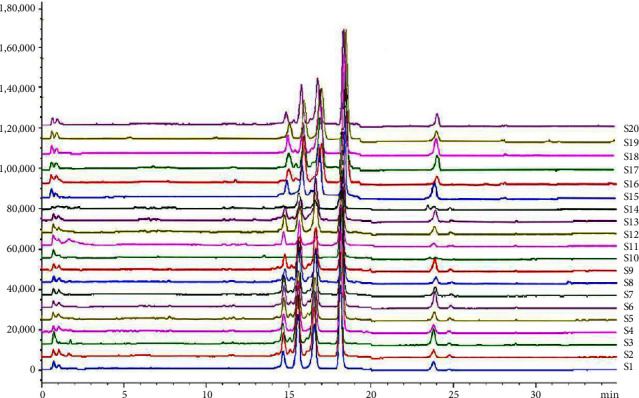
HPLC fingerprint of 20 batches of *Semen Allii Fistulosi* samples from different origins (S1: Shanghai; S2: Changchun, Jilin; S3: Penxian, Sichuan; S4: Shaoxing, Zhejiang; S5: Feifang, Shangdong; S6: Ningxian, Hebei; S7: Dalian, Liaoning; S8: Xian, Shaxi; S9: Ningyang, Shandong; S10: Lianyungang, Jiangsu; S11: Chengdu, Sichuan; S12: Urumuqi, Xinjiang; S13: Wuwei, Gansu; S14: Harbin, Heilongjiang; S15: Zigong, Sichuan; S16: Tangshan, Hebei; S17: Tianjin; S18: Bengbu, Anwei; S19: Changzhi, Shangxi; S20: Mengzi, Yunnan.

The contents (*n* = 3) of the 7 steroidal saponins of *Semen Allii Fistulosi* samples were calculated using an external standard method derived from their respective calibration curves, as listed in Table 6. Representative HPLC chromatograms are delineated in Figures 4 and 5.

### 3.3. Discussion

#### 3.3.1. Discussion for the HPLC-ELSD Method

##### 3.3.1.1. Selection of Reference Materials

Pharmacological studies have established that the steroidal saponins of *Semen Allii Fistulosi* are responsible for cardiovascular activity, with Fistulosaponin B, Fistulosaponin D, Fistulosaponin F, and Lw-24 having a higher content of components in the extract of *Semen Allii Fistulosi*. Therefore, these four steroidal saponins were selected as determination indexes for *Semen Allii Fistulosi* originating from various producing areas.

##### 3.3.1.2. Selection of Mobile Phases

The ethanol-water system was utilized in the preliminary experiment but had a poorer separation degree than the acetonitrile-water system. Consequently, the acetonitrile-water system was chosen as the mobile phase. During the experiment, the peak shape was improved by reducing the pH of the mobile phase while minimally influencing the separation degree of the chromatographic peak. Notable, optimal peak shape and separation were achieved at a pH value of approximately 3. Thus, the mobile phase is finalized as acetonitrile-water (formic acid regulates pH 3.3).

##### 3.3.1.3. Investigation of Column Temperature

Column temperatures of 25°C, 30°C, and 35°C were investigated. The results exposed that the separation degree of the four components was superior at 30°C.

##### 3.3.1.4. Investigation of Flow Rate

Different flow rates were assessed. At a flow rate of 0.8 mL/min, the peak shape widened. In contrast, at a flow rate of 1.2 mL/min, there was minimal improvement in peak shape. Considering the column pressure, the flow rate was finally set at 1.0 mL/min.

##### 3.3.1.5. Determination of ELSD Working Parameters

In the experiment, parameters such as drift tube temperature and carrier gas pressure were initially adjusted but did not yield significant improvements in the peak shape and separation degree. Therefore, the general parameters were selected as the operating parameters for ELSD, namely, a drift tube temperature of 40°C and a carrier gas pressure of 3.5 Bar. These chromatographic conditions resulted in a favorable chromatographic peak shape symmetry, with the primary components achieving baseline separation.

##### 3.3.1.6. Study on Pretreatment Conditions of *Semen Allii Fistulosi*

The dissolution characteristics of saponins are similar. Therefore, the content of the four saponins was considered in the pretreatment conditions. Three commonly used methods, namely thermal reflux, Soxhlet extraction, and ultrasonic extraction, were selected for the experiment. In the orthogonal experiment design, the ratio of solvent consisted of pure water, 30% methanol, and 50% methanol. Solvents over 50% ethanol were not utilized, given that the saponins used in this test had higher water solubility and lower alcohol solubility. The experimental results corroborated that pure water was the most effective for the extraction of the four saponins.

##### 3.3.1.7. Sample Analysis

Table 3 summarizes the content of the four active components of 13 batches of *Semen Allii Fistulosi* originating from different regions. The results uncovered that the content of saponins in this experiment was not substantially high. Among them, Fistulosaponin B exhibited the highest content, followed by Fistulosaponin D. It is worthwhile emphasizing that the content of each component was significantly different. The differences in the content of Fistulosaponin D, B, and Lw-24 were 4.85, 2.42, and 5.90 times, respectively. These results indicated that the content of secondary metabolites fluctuated during the growth of medicinal materials due to the effects of geographical environment and climate. Of note, the contents of Fistulosaponin D and Fistulosaponin B in Shanghai were 0.185‰ and 0.353‰, respectively, which were significantly higher than those in other species. Likewise, the contents of Fistulosaponin F and Lw-24 were also relatively higher. However, the content of Fistulosaponin B and Fistulosaponin D was relatively lower, and that of Fistulosaponin F was lower than the detection limit in Xian Shaxi.

#### 3.3.2. Discussion for the HPLC-MS Method

In the mixed chromatogram of the control substance (Figure 4), the chromatographic peaks of the seven steroidal saponins were completely separated within 30 min under the optimized chromatographic conditions, demonstrating a short separation time and effective separation. Table 6 showed that except for the content of Fistulosaponin E in *Semen Allii Fistulosi* samples from Shanghai being below the detection limit, the remaining samples could be quantitatively determined. These results unveiled that the content of Fistulosaponin A, Fistulosaponin C, and Fistulosaponin E was very low, and that the total content of the detected saponins was also not high in the medicinal materials, highlighting the necessity to pretreat the medicinal materials for the treatment of MI. The total saponin content in samples from Penxian, Sichuan Province, was the highest, reaching 739.4 *μ*g/g, while that from Mengzi, Yunnan Province, was 732.8 *μ*g/g. At the same time, the total saponin content in samples from Shanghai, Tianjin, and Bengbu, Anhui Province were 639.8 *μ*g/g, 548.8 *μ*g/g, and 528.0 *μ*g/g. The total saponin content in samples from Urumqi, Xinjiang Province, was 520.4 *μ*g/g. The lowest content of saponins was 222.5 *μ*g/g from Xian, Shaxi Province, with a 3.3 times difference between the highest and the lowest content. This finding suggests that the geographical environment, climate, and other factors can significantly alter the levels of secondary metabolites. Therefore, strict adherence to planting environment and GAP standards is essential to ensure the stable quality of produced medicinal materials.

As pharmacological studies have shown that the steroidal saponins were active components of *Semen Allii Fistulosi* to prevent and treat MI. It was also found that the active part of the extract of *Semen Allii Fistulosi* mainly contains 10 saponins, including the higher four saponins mentioned above, which were selected as the determination indexes in HPLC-ELSD quantitative analysis. The content of steroidal saponins in *Semen Allii Fistulosi* is relatively low and steroidal saponins usually has no UV absorption, it is difficult to determine its content by general HPLC method, and HPLC-ELSD and HPLC-MS methods are easy to do this. In the HPLC-ELSD quantitative analysis method, the chromatogram samples of *Semen Allii Fistulosi* demonstrated that under optimized chromatogram conditions, the four steroidal saponins achieved baseline separation within 40 min. Besides, the test results of the analytical methodology revealed that the method has good repeatability, high precision, and a satisfactory recovery rate. However, the content of saponins in some samples was lower than the quantitative limit, which signaled that the sensitivity was insufficient. The established HPLC-MS quantitative analysis method was used to determine the contents of 7 steroidal saponins in 20 samples of water extracts from different origins. Under optimized conditions, the saponins achieved baseline separation within 30 min, with an adequate MS response. The results portrayed that the method was rapid, sensitive, accurate, and reproducible and was ideal for the determination of steroidal saponins in *Semen Allii Fistulosi*. Both ELSD and MS are quality detectors that can be applied to detect nonvolatile compounds, including amino acids, fatty acids, sugars, and surfactants, especially compounds with no UV absorption or UV terminal absorption, such as phospholipids, saponins, alkaloids, and steroid, which are difficult to be analyzed, offering additional advantages over other HPLC detectors.

## 4. Conclusions

Quantitative determination analysis of steroidal saponins is very challenging, especially the determination of trace amount saponin content. Herein, two highly sensitive and efficient methods for the detection of steroidal saponins of *Semen Allii Fistulosi,* including minor saponins, were pioneered. The methods exhibited good linearity, precision, stability, and recovery and can be used to evaluate the quality of seeds of Allium and Allium plants. The method allows the process of quantitative determination of steroidal saponins much easier.

## Figures and Tables

**Table 1:** Regression equation, linear range, LOD, and LOQ for 4 steroidal saponins.

Components	Linear equation	*R* ^2^	Linearity range (*μ*g/mL)	LOD (*μ*g/mL)	LOQ (*μ*g/mL)
Fistulosaponin B	*y* = 1.2993*x* − 0.4147	0.9991	110.00∼1760.00	5.82	19.40
Fistulosaponin D	*y* = 1.3618*x* − 0.4576	0.9991	96.25∼1540.00	5.74	19.13
Fistulosaponin F	*y* = 1.5416*x* − 0.5731	0.9996	73.44∼1175.00	6.08	20.27
LW-24	*y* = 1.3396*x* − 0.019	0.9996	52.50∼840.00	5.25	17.50

Abbreviations: LOD, limit of detection; LOQ, limit of quantification.

**Table 2:** Precision, repeatability, stability, and recovery for the determination of the 4 steroidal saponins by HPLC-ELSD.

Components	Precision *n* = 6 interday (RSD%)	Precision *n* = 6 intraday (RSD%)	Repeatability *n* = 6 (RSD%)	Stability *n* = 6 (RSD%)	Accuracy *n* = 6 recovery % (RSD %)	
Fistulosaponin B	1.75	1.75	1.07	1.74	99.08	1.75
Fistulosaponin D	1.67	1.86	0.72	1.28	98.24	1.58
Fistulosaponin F	1.54	1.53	2.14	2.16	98.75	1.78
LW-24	2.48	2.02	2.44	2.01	99.02	1.52

Abbreviations: HPLC-ELSD, high-performance liquid chromatography-evaporative light scattering detection; RSD, relative standard deviations.

**Table 3:** Quantitative results for the 4 steroidal saponins in *Semen Allii Fistulosi* samples (*μ*g/g).

Place of origin	Fistulosaponin B	Fistulosaponin D	Fistulosaponin F	LW-24
Harbin, Heilongjiang	212.11	121.56	41.52	53.32
Wuwei, Gansu	218.09	125.53	43.08	27.82
Xian, Shaxi	146.11	66.00	Nda	12.19
Dalian, Liaoning	190.24	75.06	39.60	14.10
Changchun, Jilin	250.92	123.52	61.90	11.74
Zigong, Sichuan	171.17	156.37	37.68	31.54
Shaoxing, Zhejiang	228.58	79.90	48.90	16.68
Tangshan, Hebei	292.60	40.54	33.58	40.72
Tianjin	258.78	129.95	47.58	69.33
Ningyang, Shandong	351.92	39.95	45.80	34.98
Ningxian, Hebei	180.92	119.82	30.24	39.96
Shanghai	353.42	185.26	82.82	24.40
Chengdu, Sichuan	260.77	193.87	39.88	29.11

^a^Nd, not detected.

**Table 4:** Linear equation, linear range, LOD, and LOQ for the 7 steroidal saponins.

Components	Linear equation	*R* ^2^	Linearity range (*μ*g/mL)	LOD (*μ*g/mL)	LOQ (*μ*g/mL)
Fistulosaponin A	*y* = 0.0248*x* + 0.0026	0.9997	0.20∼7.84	0.12	0.38
Fistulosaponin B	*y* = 0.0252*x* + 0.3220	0.9996	10.86∼434.20	0.29	0.87
Fistulosaponin C	*y* = 0.0475*x* + 0.0118	0.9995	0.49∼9.84	0.27	0.88
Fistulosaponin D	*y* = 0.0556*x* + 0.5256	0.9999	7.66∼306.40	0.90	1.77
Fistulosaponin E	*y* = 0.0458× – 0.0002	0.9998	0.24∼9.48	0.13	0.45
Fistulosaponin F	*y* = 0.0393*x* + 0.1172	0.9999	4.60∼183.80	0.31	0.76
LW-24	*y* = 0.0399*x* + 0.0929	0.9994	1.55∼61.80	0.47	1.35

Abbreviations: LOD, limit of detection; LOQ, limit of quantification.

**Table 5:** Precision, repeatability, stability, and recovery for the determination of the 7 steroidal saponins by HPLC-MS.

Components	Precision *n* = 6 interday (RSD%)	Precision *n* = 6 intraday (RSD%)	Repeatability *n* = 6 (RSD%)	Stability *n* = 6 (RSD%)	Accuracy *n* = 6 recovery % (RSD %)	
Fistulosaponin A	1.45	1.13	3.24	3.70	98.90	2.41
Fistulosaponin B	1.62	1.16	3.79	1.45	97.84	0.87
Fistulosaponin C	1.21	1.38	2.96	2.53	98.17	2.02
Fistulosaponin D	2.09	1.75	3.51	0.92	96.97	1.24
Fistulosaponin E	1.77	1.39	4.33	3.47	98.48	0.88
Fistulosaponin F	1.26	0.77	4.65	2.74	96.87	1.09
LW-24	1.55	1.18	3.78	1.57	99.23	1.76

Abbreviation: HPLC-MS, HPLC-mass spectrometry; RSD, relative standard deviations.

**Table 6:** Quantitative results for the 7 steroidal saponins in *Semen Allii Fistulosi* samples (*μ*g/g).

Place of origin	Fistulosaponin A	Fistulosaponin B	Fistulosaponin C	Fistulosaponin D	Fistulosaponin E	Fistulosaponin F	LW-24
Shanghai	0.44	363.97	1.24	168.98	Nda	77.79	27.40
Changchun, Jilin	0.71	267.13	6.10	133.12	1.12	65.69	9.94
Penxian, Sichuan	4.24	444.34	8.36	160.72	4.34	86.46	30.96
Shaoxing, Zhejiang	0.90	211.53	4.07	76.51	1.38	50.52	16.78
Feifang, Shangdong	1.58	180.61	5.89	41.01	2.60	39.48	10.19
Ningxian, Hebei	3.66	311.58	5.59	79.92	2.16	47.45	53.04
Dalian, Liaoning	0.97	188.66	1.85	63.18	0.53	38.13	10.86
Xian, Shaxi	0.63	140.72	1.98	57.22	0.55	7.80	13.15
Ningyang, Shandong	2.33	304.68	2.40	42.14	1.50	47.21	38.97
Lianyungang, Jiangsu	1.22	111.21	3.12	89.03	0.55	42.37	1.58
Chengdu, Sichuan	0.74	287.92	3.12	178.59	0.65	46.18	23.80
Urumuqi, Xinjiang	2.49	303.78	2.36	134.73	1.16	28.80	47.07
Wuwei, Gansu	1.93	244.02	3.99	124.54	1.70	44.47	37.83
Harbin, Heilongjiang	3.41	236.84	4.03	114.64	1.26	38.69	48.17
Zigong, Sichuan	6.02	207.54	4.08	167.31	3.14	40.26	27.12
Tangshan, Hebei	7.31	325.04	4.24	39.49	1.31	34.29	47.98
Tianjin	6.06	286.46	3.88	138.19	0.69	51.59	61.89
Bengbu, Anwei	3.69	369.15	3.45	49.16	1.32	75.43	25.84
Changzhi, Shangxi	6.75	360.93	3.89	65.55	1.60	49.76	28.32
Mengzi, Yunnan	7.08	404.61	14.26	171.05	3.24	73.64	58.93

^a^Nd, not detected.

## Data Availability

The data used to support the findings of this study are included in the article.
